# Bradycardia in Recent Heart Transplant: Will the Microscope Illuminate the True Answer?^CME^

**DOI:** 10.14797/PREM9909

**Published:** 2021-06-16

**Authors:** Amit Alam, Philip F Halloran, Christo Mathew, Samreen Fathima, Alexia Ghazi, Parag Kale, Shelley A Hall

**Affiliations:** 1 BAYLOR UNIVERSITY MEDICAL CENTER, DALLAS, TEXAS; 2 COLLEGE OF MEDICINE, TEXAS A&M HEALTH SCIENCE CENTER; 3 UNIVERSITY OF ALBERTA, EDMONTON, CANADA

**Keywords:** allograft rejection, endomyocardial biopsy, molecular microscope

## Abstract

Transplant recipients are at risk of developing rejection that may cause significant morbidity and mortality following transplantation The clinical presentation of rejection may be atypical, leading to difficulties in diagnosis and management especially in cases with a nondiagnostic biopsy specimen. The emergence of artificial intelligence may aid in clinical decision making when traditional techniques are inconclusive.

## INTRODUCTION

With the increased volume of heart transplants, it is crucial for providers to be aware of potential complications patients face and their inherent management challenges. One such complication is acute rejection, although improved immunosuppressive regimens in recent years have helped lower the incidence of rejection and hospitalization for heart transplant patients.^1^

## QUESTION 1

***What is the current gold standard for diagnosing rejection after heart transplantation?***

- *Cardiac magnetic resonance imaging*
- *2-dimensional echocardiogram*
- *Endomyocardial biopsy*
- *Molecular Microscope Diagnostic System*

## ANSWER 1

(C) Endomyocardial biopsy

According to the most recent International Society for Heart and Lung Transplantation (ISHLT) guidelines, the current standard of care for all adult orthotopic heart transplant (OHT) recipients is to undergo periodic endomyocardial biopsy (EMB) 6 to 12 months postoperatively for surveillance of heart transplant rejection or at any point if there is a clinical suspicion for rejection.^2^ After this time, periodic EMB surveillance every 4 to 6 months is recommended for heart transplant recipients at higher risk for late acute rejection. Beyond a period of 5 years post heart transplantation, the routine use of EMB is optional depending on clinical judgment and the risk of late allograft rejection.^2^

Although EMB is the ISHLT’s method of choice for primary assessment and surveillance when transplant rejection occurs,^2^ its utility in detecting rejection is restricted when sampling and reporting issues arise and can thus lower the sensitivity for accurate diagnosis. With nondiagnostic EMB results, physicians have newer options in place such as donor-derived cell-free deoxyribonucleic acid (DD-cfDNA) and the Molecular Microscope Diagnostic System (MMDx^TM^, One Lambda, Inc.). However, without proper guideline support for these tools, care teams must collaborate creatively to manage patients with suspicion for rejection.

## CASE HISTORY

A 56-year-old African American male with left ventricular noncompaction cardiomyopathy and ventricular tachycardia on amiodarone underwent orthotopic heart transplantation (OTH). The postoperative hospital course was complicated by intermittent asymptomatic junctional rhythm in the 50s. The patient was discharged on terbutaline after intermittent sinus rhythm improved to the 80s. His immunosuppression regimen consisted of tacrolimus, azathioprine, and prednisone. Six weeks after transplant, the patient had normal routine surveillance studies (Table 1) except for a new diagnosis of donor-derived coronary disease based on intracoronary intravascular ultrasound. Outpatient Holter monitoring showed evidence of heart rate between 35 bpm while sleeping to 80s when awake, with no evidence of pauses or patient-triggered alarms.

**Table 1.** Post-transplant testing at 6 weeks and 4 months. ABMR/AMR: antibody-mediated rejection; CAV: cardiac allograft vasculopathy; CMV: cytomegalovirus; DD cfDNA = donor-derived cell-free deoxyribonucleic acid; DSAs: donor-specific antibodies; EF: ejection fraction; EMB path: endomyocardial biopsy pathology; GEP: gene expression profiling; IVUS: intravascular ultrasound; LHC: left heart catheterization; MMDx: Molecular Microscope Diagnostic System; TCMR: T-cell-mediated rejection; 1R, pAMRO: grade 1R (mild) pathology antibody-mediated rejection


TIME POST TRANSPLANT	INDICATION	ECHO	RHC	LHC/IVUS	CMV LEVELS	GEP	DD cfDNA	DSAs	EMB Path	MMDx

6 weeks	Routine surveillance	Normal EF	Preserved hemodynamics	CAV grade 0 / IVUS grade 4	Not detected	Not performed	0.12%	Non	0R, pAMR0	No TCMR / No ABMR
4 months	Bradycardia	Normal EF	Preserved hemodynamics	Not performed	Not detected	38	0.48%	Positive	1R, pAMR0	Moderate TCMR/No ABMR


Four months after transplant, the patient was admitted for loss of consciousness. Extensive workup for seizures and COVID-19 testing was negative. Outpatient tacrolimus levels varied between 8.2 and 15.2 ng/mL prior to admission. The patient was noted to have a junctional rhythm in the 30s that required dopamine. Repeat diagnostic workup for rejection is shown in Table 1.

## QUESTION 2

***Based on the results of Table 1, what should you do next?***

- *Repeat coronary angiography and intravascular ultrasound study*
- *Implant pacemaker*
- *Multidisciplinary team approach to consider treatment for rejection*
- *Change his antimetabolite to proliferation signal inhibitor and observe heart rate*

## ANSWER 2

(C) Multidisciplinary team approach to consider treatment for rejection

In our patient, the original post-transplant bradycardia was thought to be due to prolonged amiodarone use prior to OHT and less concerning for rejection given normal post-OHT surveillance studies. While the use of EMB, donor-specific antibodies (DSAs), and gene expression profiling (GEP) are in the ISHLT guidelines for the care of heart transplant recipients, incorporation of DD-cfDNA and MMDx are new and not yet included.^2^

Without guidelines and new follow-up tests highly concerning for rejection, we elected to have an immunology multidisciplinary team approach comprised of transplant cardiologists, advanced practitioners, fellows, immunologists, pathologists, and transplant pharmacists review the studies and formulate a plan (choice C). Since DD-cfDNA implied elevated cell death and MMDx reported T-cell-mediated rejection (TCMR), we decided to treat the patient with high-dose steroids and change his antimetabolite from azathioprine to mycophenolate mofetil. Proliferation signal inhibitors are not recommended until after 6 months post-transplant, if clinically indicated (choice D).^2^ While we acknowledged that new positive DSAs were concerning, with both negative EMB and MMDx for antibody-mediated rejection (ABMR), we elected to hold off on adding plasmapheresis or intravenous immunoglobulin. With a preserved ejection fraction, stable hemodynamics, and recent angiography, a repeat catheterization would not be warranted (choice A). Implantation of a pacemaker should be considered if treatment for rejection failed (choice B).

## CASE CONTINUED

**Figure 1.**
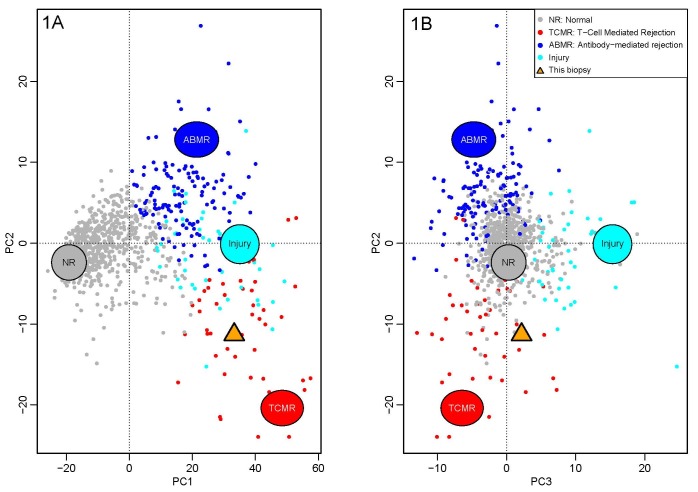
Molecular Microscope Diagnostic System report. The yellow arrow shows the biopsy. AMBR: antibody-mediated rejection; NR: normal; TCMR: T-cell-mediated rejection; PC: principal component

**Figure 2.**
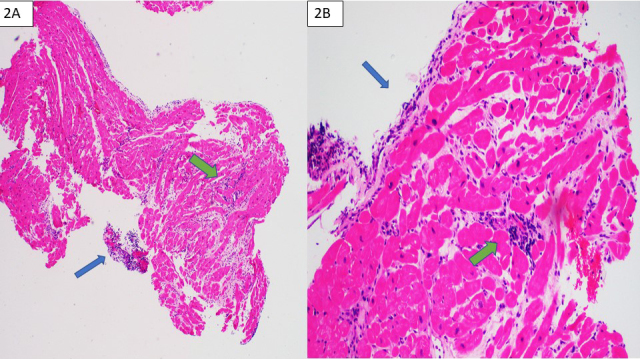
Endomyocardial biopsy.

At this time, we performed MMDx imaging (Figures 1 A, B) and EMB (Figures 2 A, B).

## QUESTION 3

***Based on the results of the MMDx (Figure 1 A, B), what is the diagnosis for our patient?***

- *Antibody-mediated rejection with injury pattern (ABMR)*
- *T-cell–mediated rejection with injury pattern (TCMR)*
- *Combined antibody and T-cell–mediated rejection*
- *No rejection*

## ANSWER 3

(B) T-cell-mediated rejection with injury pattern (TCMR)

The result from our patient’s biopsy specimen (yellow triangle) is represented among 889 reference biopsies (remaining circles) that are distributed by their molecular-rejection- related measurements in a three-dimensional data cloud.^3^ Figure 1 A shows the main variation (principal component [PC], x-axis, normal vs. abnormal) compared to the second aspect of variation (PC2, y-axis, that separates ABMR from TCMR).^3^ Figure 1 B rotates the data cloud to show the third axis of variation (PC3, separating acute injury from rejection).^3^ Thus, our patient has TCMR (Figure 1 A) with injury secondary to TCMR (Figure 1 B) without evidence of ABMR.

## QUESTION 4

***The molecular microscope diagnostic system incorporates machine learning algorithms to assign probability of disease state by isolating and analyzing which of the following?***

- *Deoxyribonucleic acid (DNA)*
- *Messenger ribonucleic acid (mRNA)*
- *Histones*
- *Ribosomal ribonucleic acid (rRNA)*

## ANSWER 4

(B) Messenger ribonucleic acid (mRNA)

MMDx isolates mRNA, measures gene expression with 99% precision using gene chips, and uses machine-learning- derived algorithms to express diagnostic probabilities of each new biopsy compared to a reference set.^4^^,^^6^ In addition, machine learning overcomes errors in sample labeling, such as those seen with biopsy diagnoses.^5^^,^^6^

## QUESTION 5

***Based on the EMB shown in Figure 2, what is highlighted by the blue and green arrows, respectively?***

- *Blue = Quilty effect, Green = severe TCMR grade 3R*
- *Blue = Quilty effect, Green = mild TCMR grade 1R*
- *Blue = Pathologic ABMR grade 3, Green = mild TCMR grade 1R*
- *Blue = Pathologic ABMR grade 3, Green = severe TCMR grade 3R*

## ANSWER 5

(B) Blue = Quilty effect, Green = mild TCMR grade 1R

The Quilty effect refers to lesions consisting of a mixture of B lymphocytes and T lymphocytes and occasionally dendritic cells (blue arrows). These lesions are dense inflammatory foci that may be seen in the endocardium of transplanted hearts. Sometimes they extend deep into the myocardium or may be large, making them difficult to distinguish from rejection. The clinical significance of Quilty lesions is not clear. Mild rejection (grade 1R) is defined as interstitial and/or perivascular infiltrate with up to one focus of myocyte injury (as seen in Figure 2 A, B, green arrow). In comparison, moderate rejection (grade 2R) infiltrates have two or more foci of infiltrate with associated myocyte injury. Severe rejection (grade 3R) is more diffuse, with eosinophils and neutrophils leading to myocyte injury. Vasculitis, hemorrhage, and edema can also be found at this level of injury.

## CASE CONTINUED

After a multidisciplinary team approach to manage the patient, including high-dose steroids, and switching his antimetabolite from azathioprine to mycophenolate mofetil, the patient’s symptoms resolved.

Ultimately, the patient was discharged in sinus rhythm without terbutaline. Outpatient heart monitoring revealed no further bradycardia. In subsequent visits, he had normal heart rates and improved surveillance studies without further changes in his medication regimen.

## DISCUSSION

The utility of artificial intelligence when used by cardiac care teams should be evaluated further. As mentioned earlier, the ISHLT guidelines recommend a few alternatives to EMB for rejection monitoring, such as gene expression profiling (eg, Allomap, CareDx, Inc.) and ventricular evoked potentials monitoring, while not recommending the use of various laboratory markers and noninvasive imaging.^2^ However, little guidance is suggested for suspected rejection when there are nondiagnostic or inconclusive biopsy results. This is especially relevant because human error plays a significant role in biopsy results. One study even suggests that pathologists agreed with each other on cell-mediated rejection approximately 50% of the time.^6^

Tools such as MMDx, a system that uses machine learning to compare gene expression to a given data set, can assist in managing transplant rejection. Although mainly studied using kidney transplant rejection, MMDx has been shown to provide more diagnostic accuracy than histological biopsy results in kidney transplant patients with antibody-mediated rejection and more accurately diagnose results that were left equivocal with standard histology.^4^^,^^6^ Further studies should be considered involving cellular rejection in heart transplants and the role MMDx and other machine-learning technologies can have when compared to the current gold standard. This could play a vital role in supplementing or replacing the information provided by EMB.

## CONCLUSION

The use of machine-learning tools like MMDx proved to be invaluable in the case of our patient, who had no evidence rejection on EMB. With the information these resources provide, cardiac care teams can effectively impact the management trajectory of transplant patients with complications. Transplant standards should reflect the increased utility of these tools to ensure providers have more guidance when suspecting rejection when biopsy shows otherwise.
